# Novel methods to establish whole-body primary cell cultures for the cnidarians *Nematostella vectensis* and *Pocillopora damicornis*

**DOI:** 10.1038/s41598-021-83549-7

**Published:** 2021-02-18

**Authors:** James D. Nowotny, Michael T. Connelly, Nikki Traylor-Knowles

**Affiliations:** 1 Rosenstiel School of Marine and Atmospheric Science, University of Miami, 4600 Rickenbacker Causeway, Miami, FL 33149 USA; 2 Present Address: Biology Department, University of Maryland, 4094 Campus Drive, College Park, MD 20742 USA

**Keywords:** Biological techniques, Cell culture

## Abstract

Cnidarians are emerging model organisms for cell and molecular biology research. However, successful cell culture development has been challenging due to incomplete tissue dissociation and contamination. In this report, we developed and tested several different methodologies to culture primary cells from all tissues of two species of Cnidaria: *Nematostella vectensis* and *Pocillopora damicornis*. In over 170 replicated cell cultures, we demonstrate that physical dissociation was the most successful method for viable and diverse *N. vectensis* cells while antibiotic-assisted dissociation was most successful for viable and diverse *P. damicornis* cells. We also demonstrate that a rigorous antibiotic pretreatment results in less initial contamination in cell cultures. Primary cultures of both species averaged 12–13 days of viability, showed proliferation, and maintained high cell diversity including cnidocytes, nematosomes, putative gastrodermal, and epidermal cells. Overall, this work will contribute a needed tool for furthering functional cell biology experiments in Cnidaria.

## Introduction

Cell culture is a valuable tool used for growing and maintaining cells in a controlled ex vivo environment^1^. Primary cell cultures are a type of short-term cell culture where the cells being maintained are the same types that came directly from the tissue, resulting in a diverse mix of cell types^2^. Primary cell cultures of multiple cell types are particularly important for studying emerging model organisms because they allow for observation and manipulation of a diversity of undescribed cell types that are still functioning as they would in vivo^3–5^.

The phylum of Cnidaria is a diverse group of predominantly marine organisms with 2 tissue layers (ectoderm and endoderm) united by the presence of cnidocyte stinging cells^5^. They are the sister group to bilaterians, and therefore represent an important branch for our understanding of bilaterian evolution^6, 7^. Cnidarians, and in particular scleractinian corals, are also critically important for ocean biodiversity and health^43,44^. However, much about the cell biology of anthozoans is still unknown^10,11^, in part due to there being no reliable cell culture method established^12–14^.

Historically, cnidarian cell cultures have been challenging to maintain due to contamination and a lack of tailored media^7,14,15^. Cell culture methods have been developed for many different cnidarian species; however, no long-lived cultures of individual cells have been established^7,14^. Cnidarian tissues are constantly exposed to their natural environment due to relatively simple tissue organization, and this along with their mucus layer, has been hypothesized to lead to a higher association with a diverse microbiome that can overgrow a cell culture^16–18^. Given these challenges there are many areas where cell culture can be improved to achieve longevity. This includes the dissociation method, media choice, and the development of antimicrobial pre-treatments.

Here, we report the first primary cell cultures using all tissues of the model sea anemone *N. vectensis*, and the development of a novel method of antibiotic-facilitated dissociation for the adult coral, *P. damicornis*. This is also the first time that individual cells from all tissues of coral or sea anemones were shown to survive in cell culture for over 12 days. Previous cell culture studies that showed over 10 days of viability were from specific tissues, produced intact multicellular structures, or had other exceptions that make their methodology not ideal for broader use (Table 1)^5,8,9,18–32^. The goal of this experiment was to build on these previous cnidarian cell culture studies and produce reliable cell cultures that can be used as a versatile tool for live cell assays. We developed an extensive antimicrobial pre-treatment protocol to reduce microbial contamination. We tested several different medias and modified a previously developed recipe which we found was the best media to promote cnidarian cell survivability while also subduing microbe growth^31^. Using this modified media, we then monitored 123 *N. vectensis* cell cultures and 51 *P. damicornis* cell cultures. We show in both *N. vectensis* and *P. damicornis* that diverse cell types can reliably survive ex vivo for over 12 days. We also show that by using these dissociation methods, antibiotic pretreatments, and modified media that both *N. vectensis* and *P. damicornis* cell cultures have early proliferation, a high cell diversity, low rates of early microbial contamination, and consistent cell morphologies throughout the time of culturing. With these new methods, live cell methods can be developed more readily enabling the development of functional assays to better understand cnidarian cell biology.

**Table 1:** Summary of previous anthozoan cell culture publications.

Publication	Model organism(s)	Notes	Maximum viability* period
Barnay-Verdier et al. 2013	*Anemonia viridis*	Tentacle tissue only	14 days
Domart-Coulon et al. 2001	*Pocillopora damicornis*	Calcium carbonate formation	N/A
Domart-Coulon et al. 2004	*Stylophora pistillata, Pocillopora damicornis*	Multicellular isolates	2 weeks
Domart-Coulon et al. 2004	*Pocillopora damicornis*	Calcium carbonate formation	6 days
Downs et al. 2010	*Pocillopora damicornis*	Early microbial interference	N/A
Drake et al. 2018	*Stylophora pistillata*	Early microbial interference	N/A
Estephane and Anctil 2010	*Renilla koellikeri*	Cell dedifferentiation reported	10 days
Frank et al. 1994	*Dendronephthya hemprichi, Heteronexia fuscescence, Favia favus, Plexaura *sp.*, Stylophora *sp.*, Clathraria *sp.*, Parerythropodium *sp.*, Milleopora *sp*., Porites *sp.	Early microbial interference	N/A
Helman et al. 2008	*Xenia enlongata, Montipora digitata*	Calcium carbonate formation	N/A
Khalesi 2008	*Sinularia flexibilis*	Early microbial interference	N/A
Kopecky and Ostrander 1999	*Acropora micropthalma, Pocillopora damicornis, Stylophora pistillata, Seriatopora hystrix, Porties sp.*	Multicellular isolates	N/A
Lecointe et al. 2013	*Pocillopora damicornis*	Multicellular isolates	7 days
Mass et al. 2012	*Stylophora pistillata*	Calcium carbonate formation	N/A
Mass et al. 2017	Stylophora pistillata	Calcium carbonate formation	7 days
Nesa and Hidaka 2009	*Fungia *sp.*, Pavona divaricata*	Multicellular isolates	3 days
Rabinowitz et al. 2015	*Nematostella vectensis*	Ectodermal tissue layers	19 days
Reyez Bermudez and Miller 2009	*Acropora millepora*	Larvae-derived	11 days
Ventura et al. 2018	*Anemonia viridis*	Tentacle tissue only	30 days

Previous publications on anthozoan cell culture: A review of past publications that produced cnidarian cell cultures .“Notes” column indicates particular tissues preparations that were used in each study. Maximum viability is what was reported in the respective publication. “N/A” is marked for publications where viability was impossible to determine given the results. (*although the publication may report a viable time period, there was either some contamination, cell loss, or loss of cell diversity during the reported time, meaning at an unknown point the cultures were not “viable” by the standards put forth in this paper).

## Methods

### Pre-culture animal preparation

#### *N. vectensis*

Adult *N. vectensis* were maintained in glass bowls containing 0.2 μm filtered 11ppt saltwater in the dark, at room temperature. Full strength saltwater was sourced from Biscayne Bay of Miami, FL, USA and diluted using reverse osmosis fresh water to bring the final concentration to 11ppt. Animals were fed 5 days per week with freshly hatched *Artemia* (Utah Sea)*.* 50% water changes were done 3 times a week^33^. In preparation for cell culture, animals were removed from bowls and rinsed three times with “anemone gentamicin medium” (AGM) which consists of sterile 11 ppt saltwater supplemented with 10 μg/ml Gentamicin Reagent Solution (Gibco by Life Technologies)^31^ (Table S1). For 3–7 days, individual anemones were isolated in AGM with daily media changes and starved. Each animal was then individually rinsed in a 2.5 μg/ml Penicillin/Streptomycin/Amphotericin B solution (PSAb) (Sigma) in 0.2 μm-filtered 11ppt saltwater and incubated at room temperature in 2.5 μg/ml PSAb for 10 min. Animals were then transferred into individual sterile 12-well tissue culture plates (VWR, Radnor, Pennsylvania) with selected media detailed below.

#### *P. damicornis*

Colonies of *P. damicornis* coral genotype, PAN-10, were originally collected from Saboga Island, Panama in 2005 and have been maintained at Rosenstiel School of Marine and Atmospheric Sciences culturing facilities^34^. The corals were maintained in an 800-gallon semi-recirculating system being constantly supplied with 10 μm-filtered sea water and were illuminated with 60 μmol·m^-2^·s^-1^ on a 12-h light/12-h dark cycle. The corals were fed using larval AP100 dry diet powder (Ziegler) twice per week. Coral tanks were cleaned twice per week to reduce algae. Just prior to starting the cell dissociation step, coral fragments approximately 1 cm in length were rinsed for 5 min with a transfer pipet using 0.2 μm-filtered full strength seawater.

### Tissue dissociation and plating of cell cultures

#### *N. vectensis*

**Figure 1:**
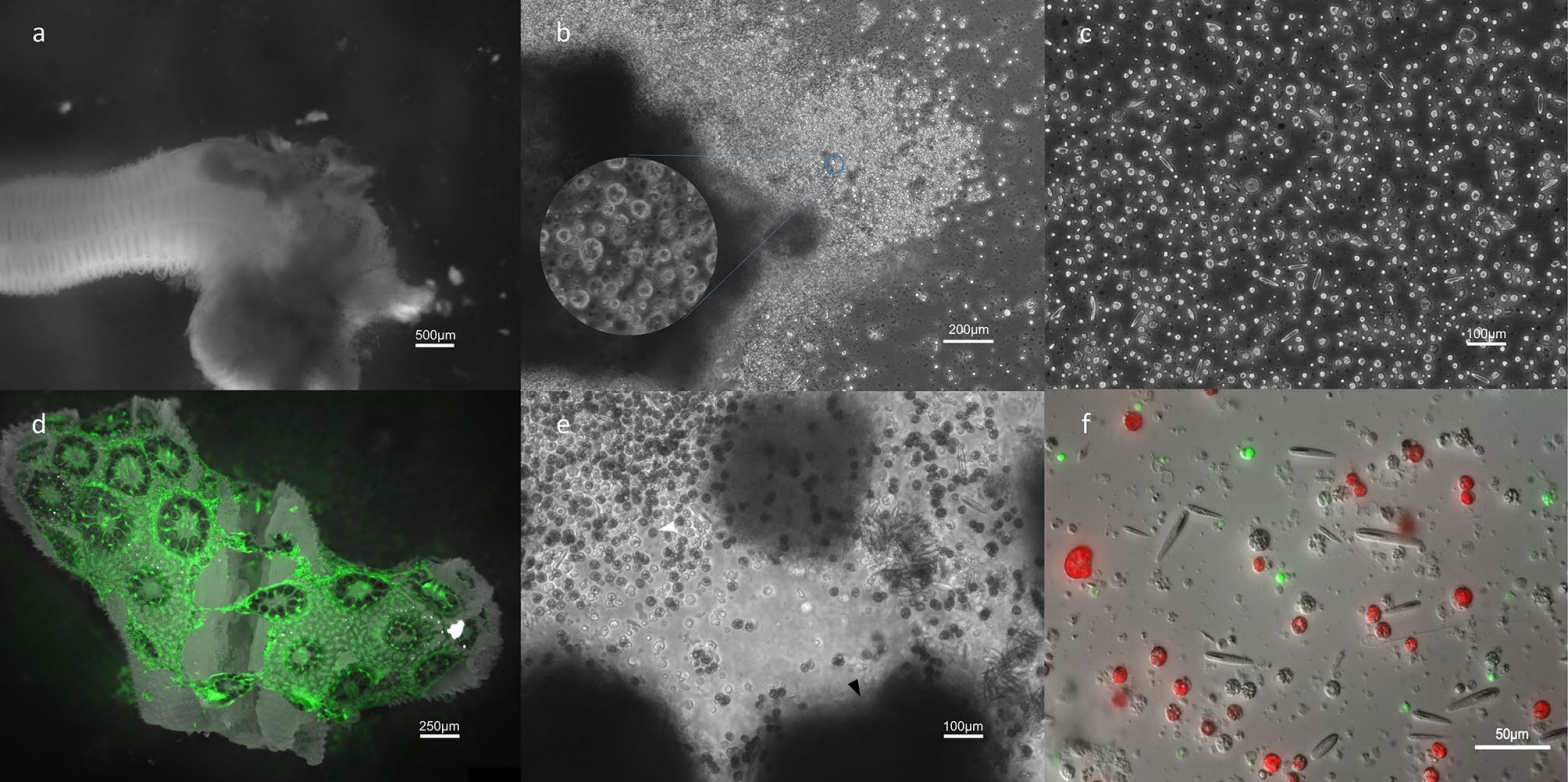
Dissociation process of *N. vectensis* and *P. damicornis* tissue. **(A)** MgCl_2_ treated *N. vectensis* with an initial scalpel cut for mechanical dissociation. **(B)** *N. vectensis* tissue clump being spontaneously dissociated into cells in culture 24 h post-dissociation (pd). **(C)** Diverse *N. vectensis* cell suspension after full dissociation 48 h pd. **(D)** Green autofluorescent *P. damicornis* tissue being sloughed off of the skeleton 8 h into antibiotic-facilitated dissociation. **(E)** *P. damicornis* cells immediately after dissociation with intact bailed-out polyps (black arrowhead) and abundant zooxanthellae (white arrowhead). **(F)** Autofluorescent *P. damicornis* cells 12 h post-dissociation with various cell types. Red fluorescence indicates algal symbionts, Symbiodiniaceae.

**Figure 2:**
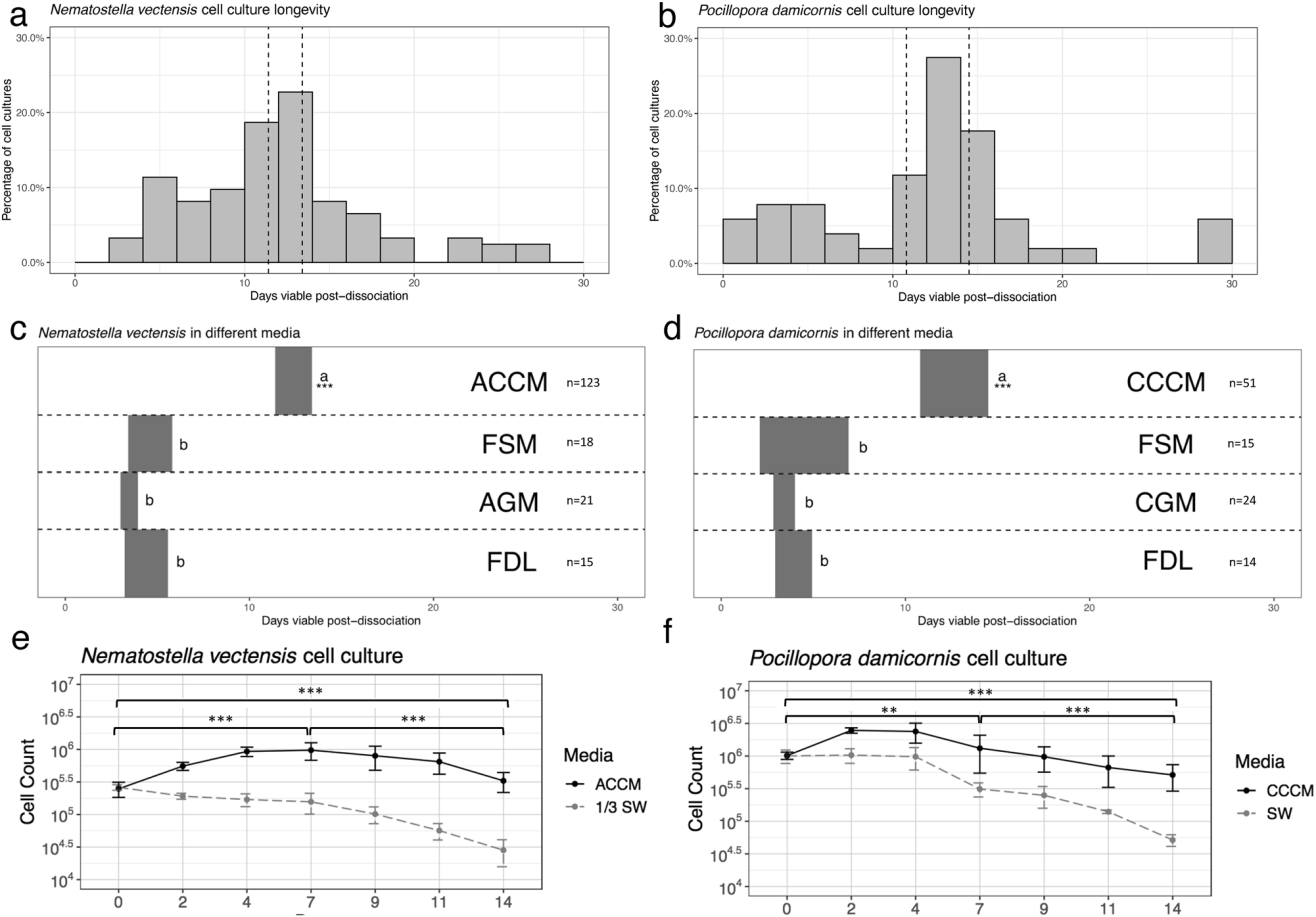
Cnidarian cell culture growth and viability. **(A)** Distribution of *N. vectensis* cell culture longevity over 123 cell cultures (n = 123, binwidth = 2 days). The dashed vertical lines indicate the 95% confidence interval after a one sample t-test (11.41879, 13.39421). **(B)** Histogram of distributions of *P. damicornis* cell culture longevity over 51 cell cultures (n = 51, binwidth = 2 days). The dashed vertical lines indicate the 95% confidence interval after a one sample t-test (10.79539, 14.53794). **(C,D)** Comparisons of viability between culture media in *N. vectensis* and *P. damicornis*. Each grey rectangle represents the 95% confidence interval for viable days in culture for all recorded cell cultures using the listed media. Two-tailed t-test between each medium were performed, a and b represent significantly different intervals (*** = P < 0.001) (*ACCM* anemone cell culture medium, *CCCM* coral cell culture medium, *FSM* full strength medium, *AGM* anemone gentamicin medium, *CGM* coral gentamicin medium, *FDL* fish disease lab media, see supplementary figures for media formulae). (**E**,**F**) Comparison of cells counts for *N. vectensis* and *P .damicornis* maintained in seawater (11ppt seawater for *N. vectensis*) or cell culture media over a 14-day period. Cell counts were plotted as log10 in cells/well of culture against time (in days). Statistical significance was determined using a non-parametric Kruskal–Wallis between counts on days 0 and 7, 7 and 14, and 0 and 14 for both species. (***P < 0.001, **P < 0.01).

**Figure 3:**
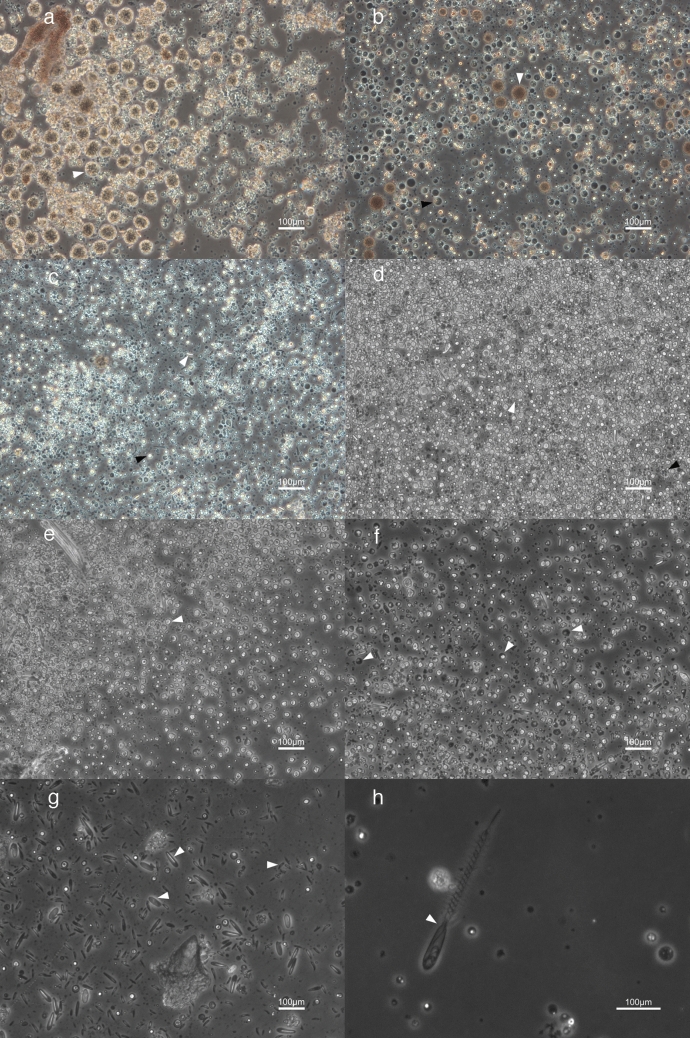
Cnidarian cell culture cell type diversity. **(A)** *N. vectensis* cells 48 h pd with several intact nematosomes (white arrowhead). **(B)** *N. vectensis* cells isolated only from the mesenteries 6 days pd. This cell suspension had mostly granulated round cells with dark vacuoles (black arrowhead) and large, occasionally mobile round clusters (white arrow). **(C)** Cells isolated only from the oral region 6 days pd. A diverse suspension of unidentified smaller round ectodermal cells (black arrowhead) along with cnidocytes (white arrowhead). **(D)** Proliferative *N. vectensis* cell culture 7 days pd with diverse cells of various shapes and sizes, including “pointed” round cells (white arrowhead) and globular cells, mostly unidentifiable based on morphology. **(E)** *P. damicornis* cell culture 21 days pd with low zooxanthellae counts, giving an observable variety of mostly unidentified host cnidarian cells in culture such as abundant rounded cells (arrowhead) **(F)** Diverse *P. damicornis* culture 25 days pd with smaller putative digestive round cells (arrowhead). **(G)** *P. damicornis* culture 28 days pd with 3 types of viable cnidocytes (arrows) (along with a piece of coral aragonite in frame) **(H)** *P. damicornis* culture 6 days pd with a large discharged cnidocyte (arrow).

We tested several different methods of dissociation including mechanical, antibiotics-facilitated with 3% PSAb, and chemical (2% *N*-acetyl l-cysteine and trypsin). Of these we found that mechanical dissociation generated the most viable cells (Figure S1). For mechanical dissociation, individual anemones were treated with 7% MgCl_2_ in 0.2 μm-filtered 11ppt seawater and then sliced with a scalpel into tissue clumps. In some cases, the tentacle tissue and mesenterial tissue were separated with the scalpel before dissociation. This was done to demonstrate different localized cell populations with pictures (Fig. 3), however, the resultant cultures were not quantified in our study. These tissue clumps were then further dissociated by repeated pipetting using a wide bore 100 μl pipet tip to reduce cell damage. Following dissociation, cell slurries from one whole organism were concentrated into 1 ml and centrifuged three times at 100×*g* for 3 min, replacing supernatant with Leibovitz’s L-15 media between each centrifugation. The resulting pellet was loosened with gentle pipetting. Then 200 μl was added to 4–5 wells of a 6-well plate with 6 ml of anemone cell culture media (ACCM) or 100 μl was added to 9–10 wells of a 12-well plate with 2.5 ml ACCM. Wells without cells were used as controls to test for media contamination. The remaining clumps were left to incubate in the media and over 24 h spontaneously dissociated to individual cells (Fig. 1A). ACCM is a modified recipe of a previously published media for *N. vectensis* ectodermal tissue culture^31^. ACCM consists of 80% AGM, and 20% full strength media (FSM), which is 95% L-15 Medium, 3% FBS, 1% PSAb, and 1% HEPES Buffer. 1% Penicillin–Streptomycin-Amphotericin b (PSAb) was also added to each well along with 7.5 μg/ml Plasmocin Prophylactic(InvivoGen, San Diego, California) to reduce bacteria growth. Other media were tested, but survival rates of cells were not as high as ACCM (Fig. 2E).

#### *P. damicornis*

Building on previous methods for dissociation of coral embryos, antibiotic-facilitated dissociation was used to cause expulsion of coral tissue from the adult skeleton^32^*. P. damicornis* fragments were submerged in 5–10 ml of antibiotic solution (enough to completely submerge the fragment): 2% PSAb, 7.5 μg/ml Plasmocin Prophylactic, and 30 μg/ml Gentamicin in 0.2 μm-filtered seawater in 6-well cell culture plates. The fragments incubated in this solution for 24–48 h to prevent microorganism growth and to promote the expulsion of the coral tissue from the coral skeleton (Fig. 1D). This treatment caused the sloughing off of live cells and “polyp bail-out”, which has been previously described in *P. damicornis* as a response to hypersalinity and toxicants^35–37^. Cells were confirmed to be viable with a 10% Trypan Blue (Gibco) exclusion test. Following antibiotic-facilitated dissociation, cells and the remaining skeletal fragments were plated in coral cell culture media (CCCM) that consisted of 20% FSM, 80% full strength 0.2 μm-filtered seawater with 10 μg/ml Gentamicin (CGM). After 5 days, the skeleton fragments were removed from culture wells with sterilized forceps. Cultures were further supplemented with 1% PSAb and 7.5 μg/ml Plasmocin Prophylactic.

### Cell culture maintenance and viability

After 1 week, media and antibiotics were changed for *N. vectensis* and *P. damicornis* cell cultures. Following this, media and antibiotics were changed every 10 days. Cultures were observed under a microscope daily for the first week and twice a week for the remaining time of the experiment. A cell culture was deemed not viable if (1) any microbial proliferation was occurring, excluding Symbiodiniaceae, (2) if fewer than ~ 100,000 living cells remained, or (3) if more than 5% of the total cells were necrotized or fragmented. Microbial proliferation was determined visually (Fig S3). We recorded when and why each cell culture lost viability, and recognized contaminants were also recorded (Fig. 2A,B, S2).

### Cell counts and viability test

Cell culture viability and cell number were recorded starting at 5 h post cell culture establishment. A 1 mL sample of cells was pipetted out of a culture and into a 1.5 mL Eppendorf tube. The contents were then centrifuged at 100×*g* for 3 min and the supernatant was replaced with a working concentration of Trypan blue stain (0.4%, Gibco Life Technologies). The stained cells were then transferred to a Reichert 0.1 mm deep improved Neubauer hemocytometer and total unstained cells were counted. These counts were performed in triplicate 3 times per week for the first two weeks in both species.

## Results and discussion

### Dissociation method affects long term cell culture viability

A total of 123 *N. vectensis* cell cultures and 51 *P. damicornis* cell cultures were tested under identical parameters and monitored for the length of their viability (Fig. 2A,B). The overall viability of the cultures was heavily dependent on the effectiveness of the tissue dissociation method. We hypothesize that the high initial cell diversity in primary cultures promotes longer cell culture viability. We define cell diversity as the number of morphologically distinct cells observed in the viable culture. However, little is understood about whether different tissue dissociation methods promote the isolation of specific populations more readily.

We found that chemical dissociation was unsuccessful in *N. vectensis*, but that mechanical dissociation yielded clumps of viable diverse cell types that spontaneously dissociated into sheets of individual cells (Figure S1, Fig. 1A–C). Also, for the first time we showed that adult tissue of *P. damicornis* is effectively dissociated using an antibiotic solution (Fig. 1D–F). The antibiotic solution caused tissue to slough off of the skeleton and after 24 h yielded a diversity of cell types (Fig. 1D–F). The process of antibiotic-facilitated dissociation for coral cell culture is a convenient innovation due to microbial mitigation and production of diverse live cells from the intact skeleton occurring concomitantly. Dissociation methods for cnidarian cell culture have been challenging due to issues with extracellular matrix strength, large amounts of mucus, and the fragility of the cells^38,39^. Chemical dissociation methods including trypsin and *N*-acetylcysteine yielded fewer viable cells, and fewer cell types due to incomplete dissociation (Figure S1). The development of the dissociation methods here are a promising breakthrough for cnidarian cell culture because they yield diverse cell types that successfully adapt to the cell culture environment quickly.

### Primary cell cultures from *N. vectensis* and *P. damicornis* are viable for 12 days on average

Of the cell culture replicates produced, 54% of *N. vectensis* and 55% of *P. damicornis* cell cultures remained uncontaminated and maintained diverse cell types for > 10 days (Fig. 2A,B). On average, *N. vectensis* cell cultures survived for 12.3 days, while *P. damicornis* cell cultures averaged 12.7 days. This represents the first time that dissociated cells from combined tissues of a sea anemone or coral have been able to survive in cell culture for over 12 days without contamination. Here we used high replication and well-defined standards for viability, which we believe translates directly into “usability” for future live cell methods. Most previous studies worked with specific or intact tissues, or cultured cells in media with notable contamination.  Among those studies, the highest definitive survival of individual cells derived from whole-body tissue appeared to be a maximum of 10 days^24^. The relatively low amount of early contamination in our cultures may be attributed to the importance of adding antibiotics at higher concentrations, as well as, the extensive rinsing and pre-treatment with antibiotics before dissociation. We also hypothesize that diluting the media to 20% allowed the cnidarian cells to outcompete any present microbes for longer periods of time. This is notable when compared to other non-diluted media tested where rapid contamination lowered viability time (Fig. 2C,D). Early contaminations were still observed on occasion, however survival up to 30 days in 3 *P. damicornis* cultures and 28 days in 3 *N. vectensis* cultures were observed (Fig. 2A,B). Why there is such variation in the sustainability of these cultures is still not understood, but differences in individual animal microbial communities could be one reason for this, however this hypothesis would need to be tested^40,41^.

Cell counts and microscope observations were used to determine if cultures were proliferating and cell diversity was employed as an indicator of cell culture viability. From 2 days post-dissociation, cell counts of both species showed prominent proliferation, followed by a viable cell suspension that was maintained for the remaining 12 days (Fig. 2E,F). Over a 14-day period, a higher amount of cell viability was observed for cells cultured in ACCM or CCCM than cells cultured in 0.2 μm-filtered seawater with antibiotics (Fig. 2E,F). Based on these findings, we believe that ACCM and CCCM are positively affecting cnidarian cell growth and survival, to a much better extent than seawater only or other established media (Fig. 2C,D).

Cell culture cessation was primarily due to overgrowth of Thraustochytrids, a known group of cnidarian associated Stramenopiles and historical cell culture “saboteurs”^16,42^. The principal limitation to long term cnidarian cell culture success is the overgrowth of microbes, and antimicrobial methods should be done as to mitigate contamination, without affecting host cell diversity. Causes of cell culture viability loss are catalogued in Figures S2 and S3. General cell culture progression from start to finish in both species is summarized in Figure S4.

### A high diversity of viable cells was observed in both cultures

Using our cell culture methods, a high diversity of cells was achieved based on complete dissociation of all tissue types (Fig. 1A–F). In *N. vectensis*, specific cells or structures such as cnidocytes or nematosomes (mobile multicellular structures that contains cnidocytes and are unique to the genus *Nematostella*), could be readily identified from whole-body cultures (Fig. 1A). To further demonstrate how cells of various tissue sources could be cultured with these methods, cultures with only mesenteries and cultures with only oral/tentacle tissue were created. Putative digestive cells were isolated in cultures using only tissue from the mesenteries and small cnidocytes and epidermal cells were isolated from tentacle and oral tissue. (Fig. 3B,C). Each of these cell types were also observed in whole-body cultures and survived up to 28 days (Fig. 3D). Nematosomes generally remained mobile in culture for up to 5 days until they dissociated into almost entirely cnidocytes, revealing much about their cellular structure.

Within *P. damicornis* cell cultures, immediately following dissociation, cnidocytes and Symbiodiniaceae-containing cells were the most recognizable cell types (Fig. 1E), however diverse round and granular cell types were also abundant even up to 30 days post-dissociation (Fig. 3E). After 5 days post-dissociation, *P. damicornis* cell cultures showed a high cell diversity due to complete cell dissociation from the skeleton (Fig. 3E,F). The presence of diverse cell types, proliferation, and cell motility in both *N. vectensis* and *P. damicornis* is indicative of a viable cell culture that will be useful for short term functional assays.

## Conclusions

Previous studies using cnidarian cell culture have been challenging due to incomplete dissociation methods, cell culture media(s) that were not promoting proliferation, and high contamination, especially from cultures derived from multiple tissue layers. This work represents the first time that fully dissociated whole-body primary cells of cnidarians have been shown to proliferate, maintain cell diversity, and remain viable for an average of approximately 12 days without contamination. The high number of cell culture replications performed in this study show that these cultures can be readily produced. The reliable production of cell cultures from whole body cnidarian tissues is valuable because it allows for the study of independent functions of cells from all tissues. These cultures are useful for the observation and manipulation of cell types in the context of other cell types, or for functionally identifying a putative unknown cell type. Accessible cnidarian primary cell cultures of all tissue sources are an innovative and convenient resource that broadens research avenues for cnidarian cell biology.

## Supplementary Information

Supplementary Legends.

Supplementary Figure S1.

Supplementary Figure S2.

Supplementary Figure S3.

Supplementary Figure S4.

Supplementary Table S1.

## Data Availability

The datasets used and/or analyzed during the current study are available from the corresponding author.
