# Author Correction: Novel methods to establish whole‑body primary cell cultures for the cnidarians *Nematostella vectensis* and *Pocillopora damicornis*

**DOI:** 10.1038/s41598-021-90241-3

**Published:** 2021-05-26

**Authors:** James D. Nowotny, Michael T. Connelly, Nikki Traylor‑Knowles

**Affiliations:** 1 Rosenstiel School of Marine and Atmospheric Science, University of Miami, 4600 Rickenbacker Causeway, Miami, FL 33149 USA; 2 Present Address: Biology Department, University of Maryland, 4094 Campus Drive, College Park, MD 20742 USA

Correction to: *Scientific Reports* 10.1038/s41598-021-83549-7, published online 18 February 2021

The original version of this Article contained errors.

In Table 1, the “Notes” and “Maximum viability* period” values were incorrectly given for the Publication “Ventura et al. 2018”. The incorrect and correct values appear below.

Incorrect:

**Table:** 

Publication	Model organism(s)	Notes	Maximum viability* period
Ventura et al. 2018	*Anemonia viridis*	Tentacle tissue only/microbial interference	N/A

Correct:

**Table:** 

Publication	Model organism(s)	Notes	Maximum viability* period
Ventura et al. 2018	*Anemonia viridis*	Tentacle tissue only	30 days

Additionally, there were errors in the Reference List. The authors omitted the below papers, which are listed as References 43 and 44.

Knowlton, N. & Jackson, J. B. C. Shifting baselines, local impacts, and global change on coral reefs.* PLoS Biol.* **6**(2), e54. 10.1371/journal.pbio.0060054 (2008).

Bellwood, D. *et al.* Confronting the coral reef crisis. *Nature* **429**, 827–833. 10.1038/nature02691 (2004).

As a result, References were incorrectly given in the Introduction.

“Cnidarians, and in particular scleractinian corals, are also critically important for ocean biodiversity and health^8,9^”

now reads:

“Cnidarians, and in particular scleractinian corals, are also critically important for ocean biodiversity and health^43,44^”.

The original Article has been corrected.
