# Supplementary material for: Novel methods to establish whole-body primary cell cultures for the cnidarians Nematostella vectensis and Pocillopora damicornis
Source: Sci Rep. 2021 Feb 18;11:4086. doi: 10.1038/s41598-021-83549-7 (PMC7893170; doi:10.1038/s41598-021-83549-7)
Supplement: Supplementary file 1 — Supplementary Legends. [file 41598_2021_83549_MOESM1_ESM.docx]

**SUPPLEMENTARY TITLE PAGE:**

**TITLE:** Novel Methods to Establish Whole-Body Primary Cell Cultures for the Cnidarians *Nematostella vectensis* and *Pocillopora damicornis*

Authors: James D. Nowotny^1,2^, Michael T. Connelly^1^, Nikki Traylor-Knowles^1^

^1:^ University of Miami, Rosenstiel School of Marine and Atmospheric Science, 4600 Rickenbacker Causeway, Miami, Florida, 33149, USA

^2:^ Current address: University of Maryland, Biology Department, 4094 Campus Drive, College Park, Maryland, 20742, USA

Corresponding author: Nikki Traylor-Knowles, University of Miami Rosenstiel School of Marine and Atmospheric Science, 4600 Rickenbacker Causeway, Florida, 33149, USA,

Email: ntraylorknowles@rsmas.miami.edu

ORCID ID: 0000-0002-4906-4537

Keywords: cell culture, Cnidaria, coral, sea anemone, invertebrate cell culture

**SUPPLEMENTARY INFORMATION**

**Figure S1:** Average initial viable cell counts from 1 whole *N. vectensis* using 4 different dissociation methods (*** = P < 0.001). Significance was determined with a two-way ANOVA test and post-hoc Tukey test between each dissociation method. Error bars indicate standard deviation. For sample size, n=42 for mechanical dissociation, n=24 for all others.

**Figure S2:** Causes of cnidarian cell culture viability loss. (A-B) Percent total of cell culture death or host contamination in *N. vectensis* (123 cultures) and *P. damicornis* cell cultures (51 cultures). Cell death was defined as uncontaminated cnidarian cells that lost viability based on trypan blue assay.

**Figure S3:** Pictures of common cell culture contaminants. (A) *P. damicornis* cell culture 17 days pd completely taken over by Thraustochytrid organisms adhered to the plate with visible ectoplasmic filaments (arrow). (B) Thraustochytrid takeover of a *N. vectensis* cell culture 36 days pd. (C) Thraustochytrids in their mobile multinucleate amoeba-like phase from an *N. vectensis* culture 21 days post dissociation. (D) Aggregate fungus with spindle-like cells with a complete takeover of a *P. damicornis* culture 18 days pd. (E) Thick layer of suspended bacteria overlaying an *N. vectensis* culture 11 days pd.

**Figure S4: Representative pictures of the progression of *N. vectensis* and *P. damicornis* cell cultures based on qualitative observations.** (A) A labelled timeline of the observed stages of *N. vectensis* cell culture. Time estimates encompass all times post dissociation (pd). Day 0-3: Shows diverse cells that result from mechanically dissociated cell clumps in culture. Day 4-14: Shows an uncontaminated diverse cell suspension in culture two types of cnidocytes (blue and yellow arrows), a round granulated cell (green arrow), an elliptically shaped, potentially secretory cell (red arrow) and an abundant small round cell (orange arrow) along with other unknown cell types. Day 15-20: Typically, granulated mesenterial round cells (purple arrow), small abundant round cells (orange arrow) and small cnidocytes (yellow arrow) account for most surviving cell types. Day 14-∞: Stage four shows most remaining cnidarian cell types such as small round cells (orange arrow) or cnidocytes (blue arrow) necrotizing while Thraustochytrid cells (white arrow) begin to adhere and eventually overcome the *N. vectensis* cells. B)  A labelled timeline of the observed stages of *P. damicornis* cell culture post- dissociation. Day 0-2:  Diverse cell suspension after antibiotic-facilitated dissociation of *P. damicornis* tissue. Day 3-7: An initial over proliferation of Symbiodiniaceae was typically observed. (red arrow) Several cnidocyte types were also observed 2(yellow and green arrows) early in culture. Day 8-14: Cell diversity is present with three types of cnidocytes (orange, yellow, and green arrows), large granulated round cells (blue arrow), Symbiodiniaceae (red arrow). During this time, Thraustochytrid cells are typically observed in both small cystic form and larger saprobic form (white arrows). Day 15-∞:  Overabundance of Thraustochytrids observed, with large lipid-producing cyst cells (white arrow) and their small flagellated zoospores (purple arrow).
