# Supplementary figures and images for: Novel methods to establish whole-body primary cell cultures for the cnidarians Nematostella vectensis and Pocillopora damicornis
Source: Sci Rep. 2021 Feb 18;11:4086. doi: 10.1038/s41598-021-83549-7 (PMC7893170; doi:10.1038/s41598-021-83549-7)

# *N. vectensis* dissociation methods

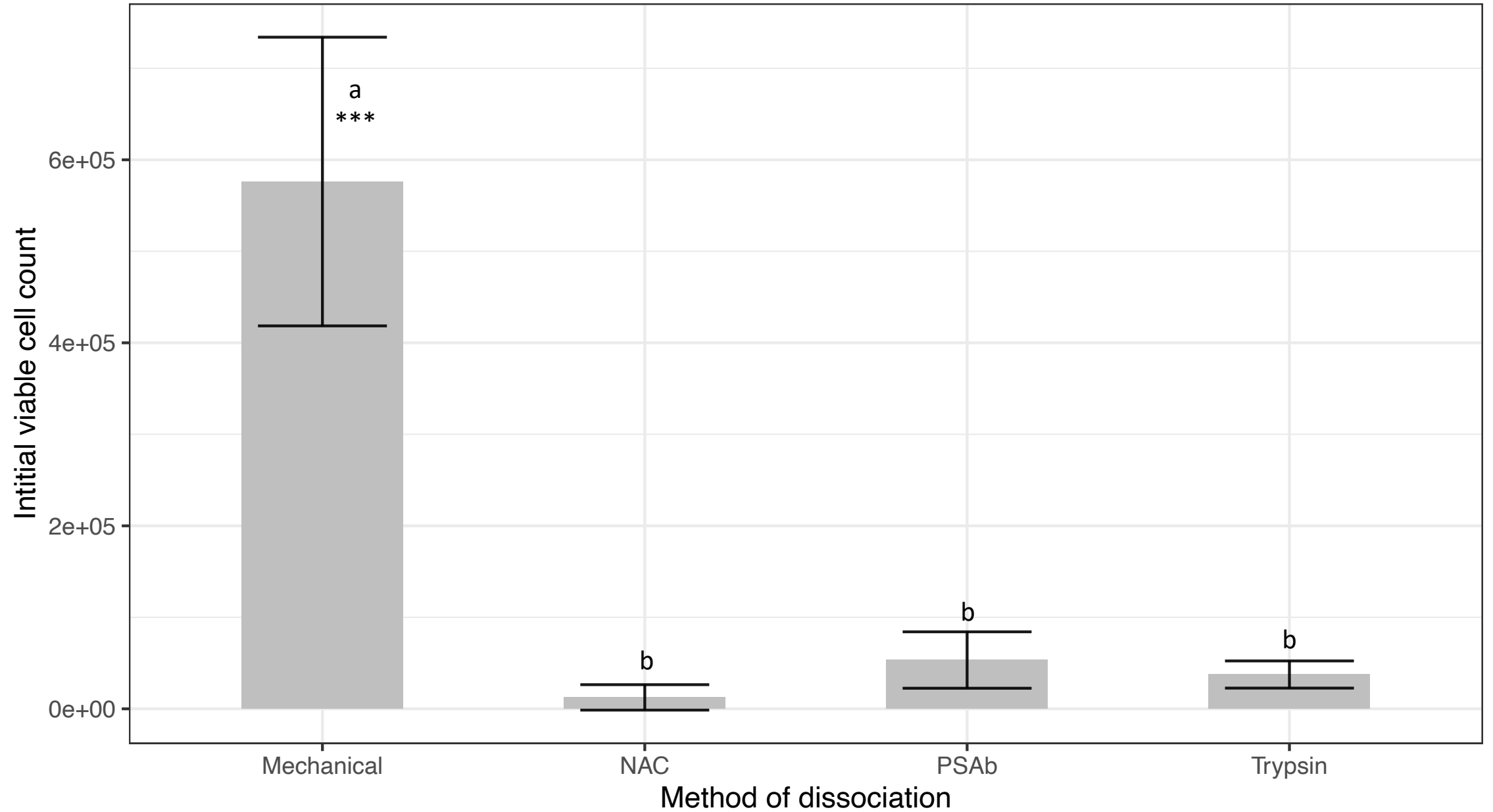

Supplement: Supplementary file 2 — Supplementary Figure S1. [file 41598_2021_83549_MOESM2_ESM.pdf]

*N. vectensis*

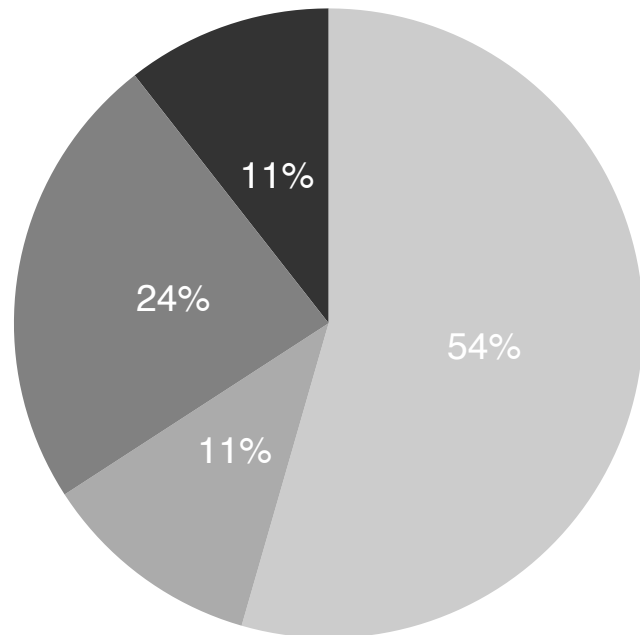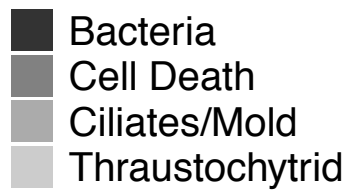

*P. damicornis*

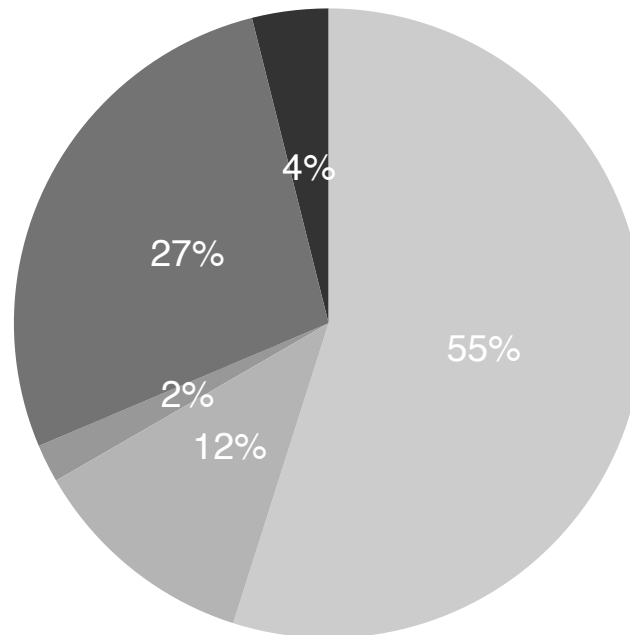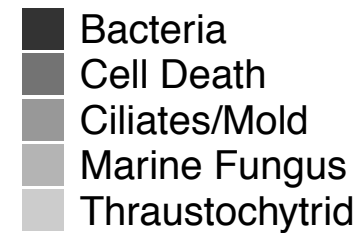

Supplement: Supplementary file 3 — Supplementary Figure S2. [file 41598_2021_83549_MOESM3_ESM.pdf]

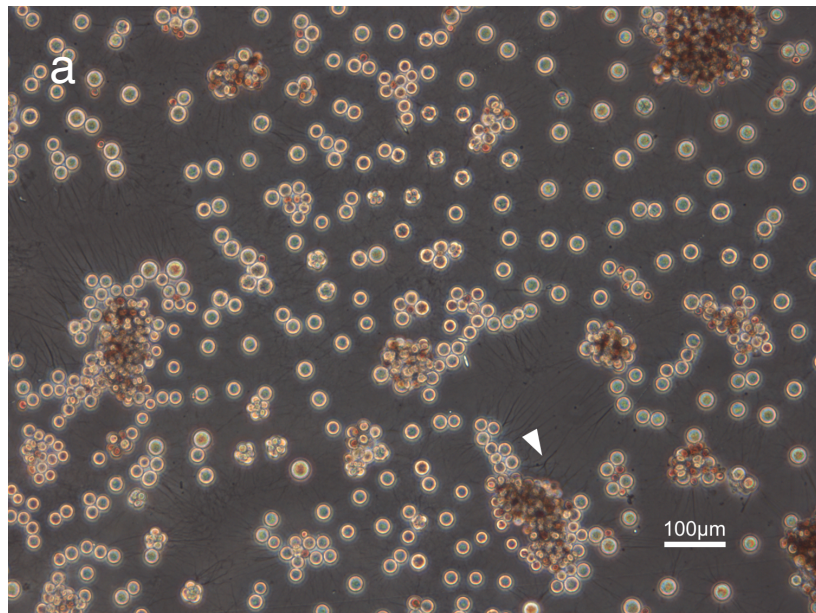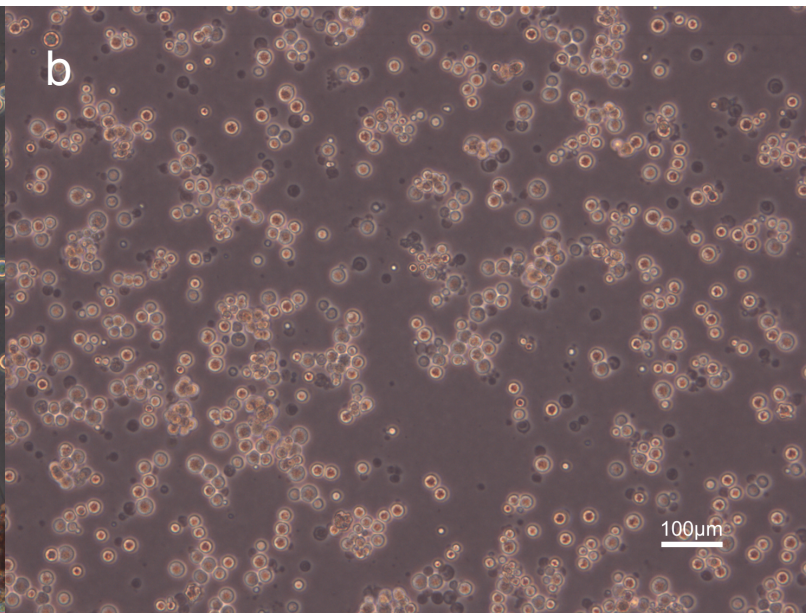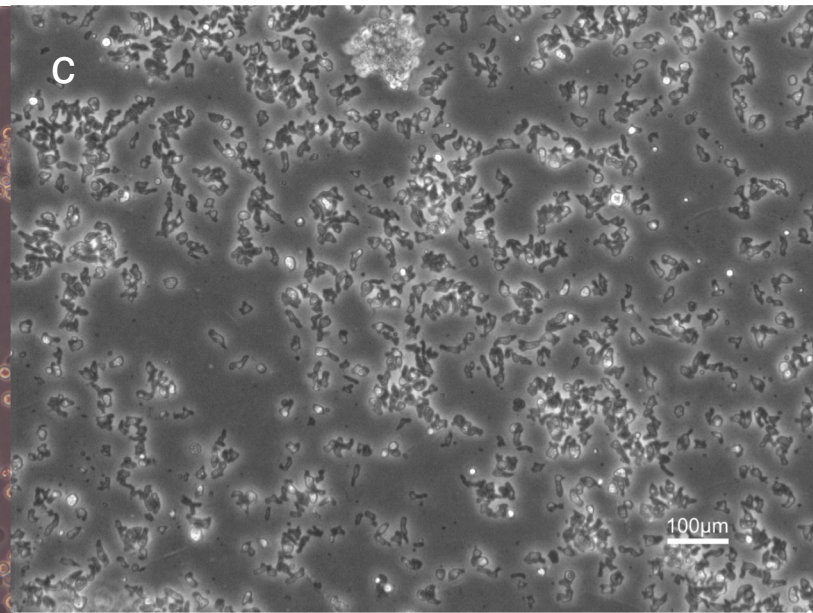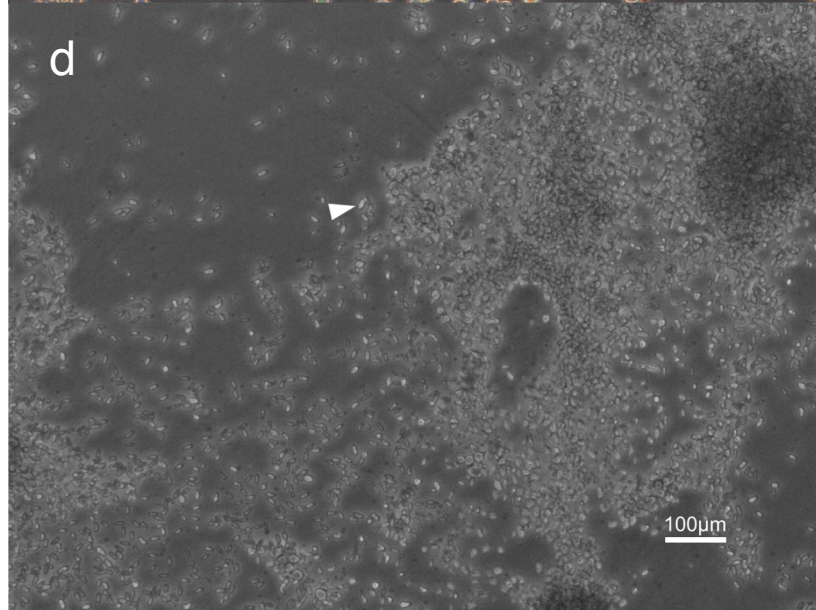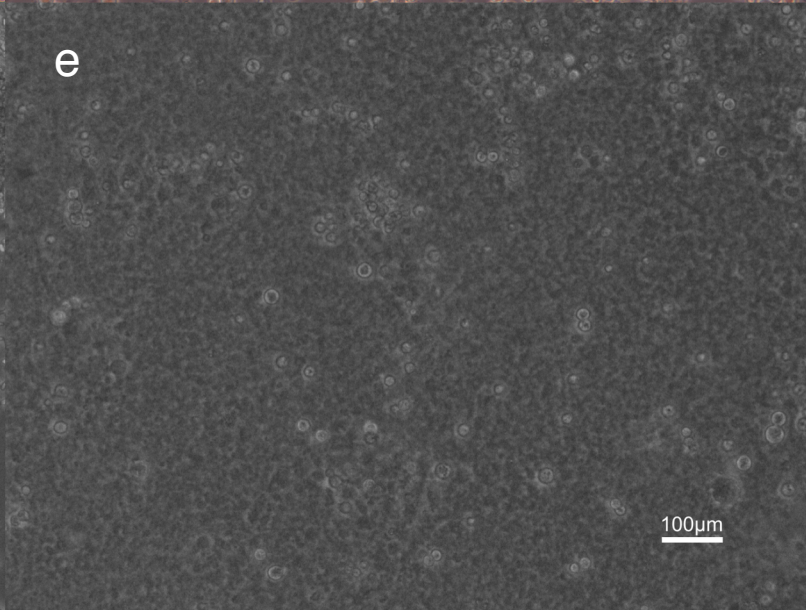

Supplement: Supplementary file 4 — Supplementary Figure S3. [file 41598_2021_83549_MOESM4_ESM.pdf]
