# Supplementary material for: Novel methods to establish whole-body primary cell cultures for the cnidarians Nematostella vectensis and Pocillopora damicornis
Source: Sci Rep. 2021 Feb 18;11:4086. doi: 10.1038/s41598-021-83549-7 (PMC7893170; doi:10.1038/s41598-021-83549-7)
Supplement: Supplementary file 5 — Supplementary Figure S4. [file 41598_2021_83549_MOESM5_ESM.pdf]

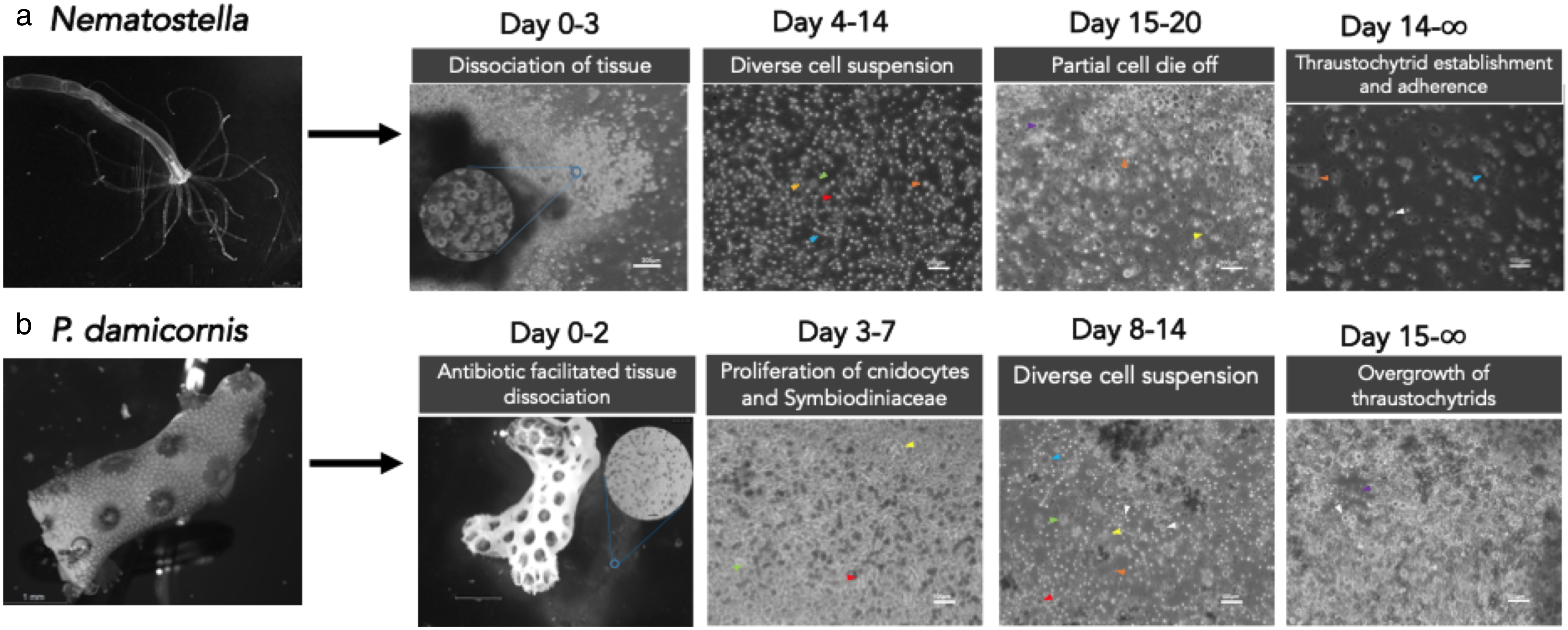

a Nematostella
Day 0-3
Dissociation of tissue
Day 4-14
Diverse cell suspension
Day 15-20
Partial cell die off
Day 14-∞
Thraustochytrid establishment and adherence
b P. damicornis
Day 0-2
Antibiotic facilitated tissue dissociation
Day 3-7
Proliferation of cnidocytes and Symbiodiniaceae
Day 8-14
Diverse cell suspension
Day 15-∞
Overgrowth of thraustochytrids
