# Supplementary material for: Novel methods to establish whole-body primary cell cultures for the cnidarians Nematostella vectensis and Pocillopora damicornis
Source: Sci Rep. 2021 Feb 18;11:4086. doi: 10.1038/s41598-021-83549-7 (PMC7893170; doi:10.1038/s41598-021-83549-7)
Supplement: Supplementary file 6 — Supplementary Table S1. [file 41598_2021_83549_MOESM6_ESM.docx]

**Table S1: Media recipes used in this study.** All percentages are by volume.

| **Name** | **Recipe** |
| --- | --- |
| Full Strength Medium (FSM) | 95% Leibovitz (L-15) Medium, 3% Fetal Bovine Serum (FBS), 1% HEPES buffer, 1% Pen/Strep/Amph b Antibiotics Solution (PSAb) (Sigma Aldrich) |
| Anemone Growth Medium (AGM) | 0.2 μm-filtered 11ppt seawater with 0.1% Gentamicin |
| Coral Growth Medium (CGM) | 0.2 μm-filtered seawater with 0.1% Gentamicin |
| Anemone Cell Culture Medium (ACCM) | 20% FSM, 80% AGM |
| Coral Cell Culture Medium (CCCM) | 20% FSM, 80% CGM |
| Fish Disease Lab Media (FDL) | 80% L-15, 8% FBS, 8% culture safe water (no Calcium or Magnesium), 1% Ampicillin, 1% PSAb, 1% 5M NaCl |
